# Development and pilot-testing of an evidence-based quality indicator set for home mechanical ventilation care: the OVER-BEAS project

**DOI:** 10.1186/s12913-024-10583-2

**Published:** 2024-01-30

**Authors:** Martha Schutzmeier, Lilly Sophia Brandstetter, Stephanie Stangl, Jutta Ahnert, Anna Grau, Laura Gerken, Hanna Klingshirn, Bernd Reuschenbach, Tobias Skazel, Maximilian Kippnich, Thomas Wurmb, Peter Heuschmann, Kirsten Haas

**Affiliations:** 1 Institute for Clinical Epidemiology and Biometry, Julius-Maximilians-University Würzburg (JMU), Würzburg, Germany; 2 Catholic University of Applied Sciences Munich, Munich, Germany; 3 Department of Anaesthesiology, Intensive Care, Emergency and Pain Medicine, Subsection Emergency and Disaster Relief Medicine, University Hospital Würzburg, Würzburg, Germany; 4 Clinical Trial Centre Würzburg, University Hospital Würzburg, Würzburg, Germany

**Keywords:** Home mechanical ventilation, Quality indicators, Quality of care, Chronic respiratory failure, Invasive ventilation, Non-invasive ventilation

## Abstract

**Background:**

The number of patients depending on home mechanical ventilation (HMV) has increased substantially in Germany in recent years. These patients receive long-term care in different nursing facilities (nursing home, shared living community, private home). However, there are limited data available on the quality of care of HMV patients. The aim of the OVER-BEAS project was to identify quality indicators (QIs) of HMV care using an evidence-based approach.

**Methods:**

A multidisciplinary board consisting of professionals and experts of HMV provision compiled a set of QIs between March and September 2019. In a structured, transparent process a set of QIs covering structures, processes and outcome of HMV patient’s care were proposed and evaluated based on the best available evidence. QIs were defined as relevant, reliable and valid measurements of the quality of HMV care and furthermore to be comprehensive and applicable in practice.

**Results:**

The experts proposed 40 QIs and consented a final set of 26 QIs. Based on the final set, questionnaires to document the QIs were developed: (1) to assess the quality and describe the structure of the nursing facility; and (2) to gather information on patient-related processes and outcomes. The feasibility of the questionnaires was tested in 5 nursing facilities treating HMV patients. The remarks from the nursing specialists were categorised in three groups: (1) term missing accuracy, (2) problem of understanding, and (3) not documented or documented elsewhere. Mean documentation time by the nursing specialists for one patient was 15 min. Based on this feedback, the questionnaires were finalised.

**Conclusions:**

We proposed a set of QIs relating to long-term HMV care and developed two questionnaires to collect this information. In a pilot study, we found the set of questionnaires to be feasible in assessing the quality of HMV care according to current evidence. The development of standardised evidence-based QIs to evaluate HMV care is a step towards implementing a standardised quality assurance program to document the quality of care of HMV patients.

**Supplementary Information:**

The online version contains supplementary material available at 10.1186/s12913-024-10583-2.

## Background

Home mechanical ventilation (HMV) is standard of care in Germany for patients with chronic respiratory failure [1]. Two types of HMV exist: 1). non-invasive ventilation (NIV), e.g. using a face mask and 2). invasive ventilation via inserting tubes/ tracheostomy [2]. For patients depending on HMV three different living situations are provided: home care (HC), specialised shared living communities (SLC), and nursing homes with a focus on long-term HMV care (NH) [2, 3]. In HC, patients live autonomous or supported by a specialised long-term care nursing service (24/7, 1:1 care). In SLC up to 12 individuals in need of care live together [3, 4]. In Germany, most HMV patients live in HC or in SLC [5]. To date, only limited evidence is available on the health care situation of HMV patients in Germany [3, 6, 7].

The number of patients depending on HMV is rapidly increasing in Germany and worldwide [8–16]. In 2005, the Eurovent survey estimated a prevalence of HMV of 6.6 per 100,000 people in 16 European countries with a total of 1,000 HMV patients in 2005 in Germany [6]. Recent surveys estimated a prevalence of HMV patients varying from 20 up to 367 per 100,000 people in different European countries [16, 17]. Currently, the number of HMV patients in Germany is estimated to be around 15,000 and 30,000 [1, 8, 9].

Population ageing and its correspondence to a higher prevalence of co-morbidities could be a reason for the rising number of HMV patients in Germany [18]. It might be caused by improving survival rate of medically dependent patient populations and widening indications as demonstrated by guidelines for invasive and non-invasive home mechanical ventilation [2], that specifically recommend that difficult-to-wean patients should be transferred to specialised weaning centres. These centres are capable of assessing the individual weaning potential and also implementing long-term, home-based care if weaning is unsuccessful.

The costs for the 1:1 care of an HMV patient at home can be up to 250.000 Euros per year [1], while the expenditure generated by out-of-hospital intensive care services is estimated at over 2.2 billion euros per year in Germany [19]. Although proven to be beneficial and cost-effective in several indications and settings [20], HMV poses a major challenge to the health system, both in respect to personnel and financial resources, as the growing need for HMV might result in a decline of quality of care and unfavourable outcomes.

Although the evidence basis for HMV and its consequences on quality of life has increased, there is still not enough data available on the quality of care of HMV patients [21]. Recently different health care professionals (HCP) were interviewed on the quality of care for patients depending on HMV [22]. The HCP mostly criticised the lack of mandatory standards and statutory quality control systems in HMV, stating that financial disincentives lead to mismanagement in the care. In 2020, the German government passed the Intensive Care and Rehabilitation Strengthening Act (IPReG) [23]. According to the IPReG, a patient’s weaning potential should be assessed regularly, access to rehabilitation should be facilitated and mandatory quality measures should be implemented [23].

However, an evidence-based standardised set of quality indicators, able to evaluate the quality of care of HMV patients in Germany, is still absent. Therefore, the objective of the present study was to develop a set of quality indicators (QIs) to measure quality of HMV care using a standardised and evidence-based approach. A second aim was to test the feasibility of these indicators in the routine care of nursing care facilities providing HMV care.

## Methods

### Constitution of the quality indicator board (QI board)

In March 2019, experts of different disciplines in the field of HMV and long-term care, working in the “Out-of-Hospital Critical Care” working group of the Bavarian State Office for Health and Food Safety (LGL), were invited to join the QI board for the development of evidence-based QIs. A total of 15 associations were reached to ensure that all relevant professions were involved in the process. The board consisted of representatives of medical long-term and emergency/intensive care (i.e. general practitioners, pulmonologists, anaesthesiologists), stakeholders from the health care professions (nursing specialists, occupational therapists, speech therapists, physical therapists), an HMV patient representative, representatives from home respiratory services, weaning centres, health insurance companies, the medical service of the health insurance companies (MD Bavaria), and methodological specialists.

### Methodological approach

The process for the development of QIs was adapted from the recommendations of the First Scientific Forum on Assessment of Quality of Care and Outcomes Research in Cardiovascular Disease and Stroke of the American Heart Association and the obligations for clinical performance measures in the German Health Care system [24, 25]. The same approach was already used in previous studies in acute and rehabilitation stroke care, as well as in Parkinson’s disease [26–28]. The approach included a standardised Delphi process, a literature search and the critical evaluation of the available evidence.

### Definition of the QI term and methodological requirements

A QI should reflect the standard of care for all appropriate patients. Thereby, a QI should include exact criteria for the selection of suitable patients for the specific indicator. Evidence-based guidelines can and should support the development of QIs [24, 25].

The consented QIs should be a relevant, reliable and valid measurement for the quality of care, as well as comprehensible and applicable in practice. Hence, the QI board agreed on the following methodological requirements for the QIs [24, 25]:

- The proposed QIs should represent an important outcome for the patient or society. If not, it must at least be closely related to those outcomes;
- It should be valid and reliable, ensuring a valuable measure of health care quality;
- It should be possible to adjust for patient variability. Observed differences should only relate to performance or process differences of the participating institutions and not disparities in patient characteristics;
- The collection of data in the long-term care setting should be feasible in the routine of health care providers;
- The QIs should be adjustable to changes in health care processes, motivating health care providers to improve their services.

### Covered health care dimension

The QI board chose the Donabedian concept as theoretical framework. According to the concept, information on quality of health care can be measured by three main dimensions: structure, process, and outcome [29]. For a better reflection of different health care dimensions in HMV, three different health care components were added to the health care dimension: 1). medical care, 2). nursing care and 3). therapeutic-rehabilitative care. The board aimed to propose at least one QI in each of the defined health care dimensions for each component of care, if applicable.

### Proposal of the QIs and evaluation of the quality of scientific evidence

In a first step, the QI board was divided into three groups according to the components of care, as mentioned previously. Each group suggested potential QIs and then provided a standardised report. This report included the covered component of health care quality; the references used (original publications, guidelines and consensus statements); the definition of suitable patients for whom the QI is valid; a possible adjustment for patient variability, if applicable; and the way of data report (e.g. rate or proportion). In a second step, the whole QI board reviewed and consented on the proposed preliminary QI set.

Following the proposal of QIs, a literature search was performed in MEDLINE (via PubMed), in the Cochrane Library and in BMJ for each of the QI. The identified evidence was prioritised by its level of evidence and its methodological quality. If no scientific evidence was available, consensus recommendations or expert statements were considered.

### Evaluation and consent of the QIs

In a third step, the QI board evaluated the preliminary QI set according to the predefined methodological requirements and discussed which QIs should be included in the final set.

In a next step, the members of the QI board rated all potential QIs in an online survey regarding their relevance and practicability. The rating ranged from 0 (QI should be removed, low/ no relevance or practicability) to 4 (high relevance or practicability). To be eligible for the final set, a QI needed to score on average at least 2 points or higher. Based on this rating, the final QI set was defined.

### Development of questionnaires and pilot study

Based on the final QI set, questionnaires for their collection in practice were developed. A prospective pilot study was implemented to further improve the quality of the questionnaires and to investigate the practicability of data collection in routine care. Facilities located in Bavaria (Germany), where HMV (invasive and non-invasive) patients lived were invited to take part in the pilot study. Information from the patient files was used to fill out the anonymous questionnaire by the nursing staff. If the information was missing or not documented in their routine, the participating facilities were asked to leave a comment in the questionnaires. Furthermore, the facilities were requested to give feedback on the questionnaires and its feasibility in their daily routine and the time needed to complete the questionnaires. On the basis of the feedback that we received from the participating nursing care facilities, the questionnaires were adapted and finalised.

### Ethics approval and consent to participate

The study was performed in accordance with the Declaration of Helsinki and all methods were fully approved by the ethics committee of the Medical Faculty of the University Würzburg (No. 57/19-sc). The study protocol approved by the ethics committee comprised that the obtainment of informed consent for the pilot study was not necessary, as all questionnaires were anonymous and based on retrospectively collected routine data and identification of patients was not possible. The requirement for informed consent was waived by the ethics committee of the Medical Faculty of the University Würzburg because of the retrospective nature of the study. The responsible data protection officer accepted the data management concept.

## Results

### Development of a set of QIs

**Fig. 1:**
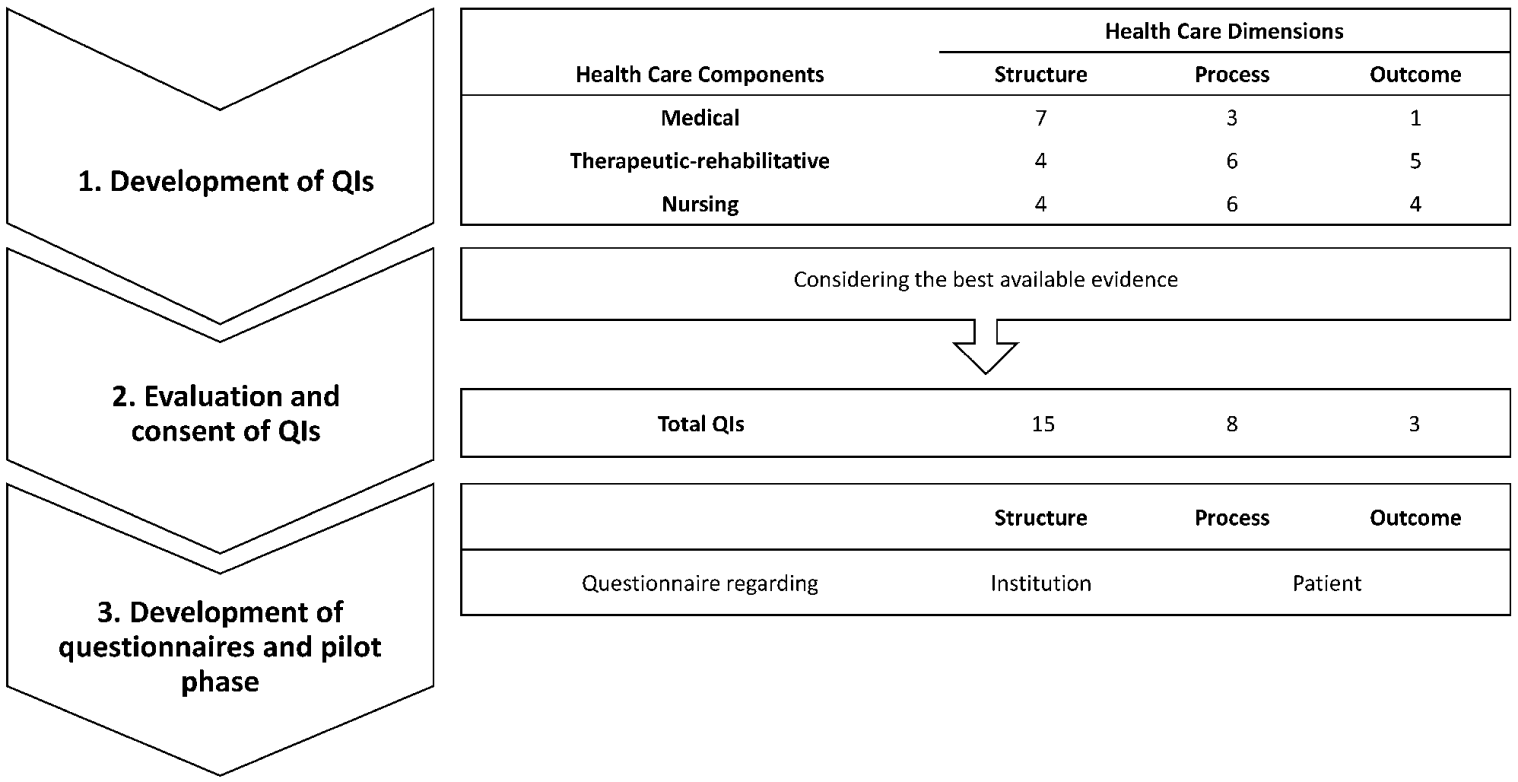
QI development process

The process to define the QI set took place in one face-to-face workshop, one phone conference and an online survey between March and September 2019. The detailed process is presented in the Fig. 1. A literature search was carried out before the QI development started. No published QIs for HMV care in Germany were identified.

At the face-to-face workshop, 40 QIs were proposed and formulated for the initial QI set. After evaluation of the scientific evidence and a consent process, 26 QI were maintained in the final QI set. The initial QI set in comparison with the final QI set is provided in the Supplement (Table S1). Of those 26 QIs 15 were classified as structural indicators, 8 as process indicators and 3 as outcome indicators. The final QI set is provided in Table 1.

**Table 1:** Overview of the final quality indicator (QI) set

Quality indicators (n = 26)		Definition	Health care component	Evidence
Structural indicators (n = 15)	Comprehensive transition management to weaning centre (from hospital)/ contact to weaning centre/ contact person at weaning centre (S_01)	Reduction of complications within the transition process	Medical	recommendation S2k-guideline
	Acute-hospitalisation of patients (S_02)	Assessment of structured transition of patients in long term care into a hospital	Medical	recommendation S2k-guideline
	Emergency management and concepts in case of infrastructure deficiencies (S_03)	Assessment of the availability contact dates of relatives, general practitioners, pulmonologist, weaning centre and medical device providers	Medical	recommendation expert
	Early detection of complications (S_04a) and Complication management (S_04b)	Reduction of complications in case of an emergency for relatives, patients, nursing care facility, other health care providers	Medical	recommendation S2k-guideline
	Qualification of nursing staff (vocational training and training for intensive care ) (S_05)	Guarantee of high quality of care; assessment of the education of the nursing staff and if necessary qualifications for the treatment of mechanical ventilated patients are obtained	Nursing	recommendation S2k-guideline
	Nursing staff to patient ratio (ambulatory intensive care - assisted living communities) (S_06)	Guarantee of a professional work environment by adequate staffing; assessment of the numbers of employed nurses and of the patients	Nursing	recommendation expert
	Qualification and additional qualification of therapists (S_07)	Standard health care; therapeutic measures should only be performed by therapists with permission to own this professional title in Germany	Therapeutic-rehabilitative	recommendation expert
	Participation of the nursing facility in external quality circles (S_10)	Guarantee of high quality of care by regular training; assessment if the nursing facility takes part in external boards and if they are in regular exchange with other facilities, thereby stays up-to-date regarding recent changes and demands and strives to improve to own quality of care	Nursing	recommendation expert
	Interdisciplinary case conferences (S_11)	Assessment of regularly performed and documented multidisciplinary (at least: pulmonologist, general practitioner and breathing therapist) case conferences	Medical	recommendation expert
	Hygiene plan and concept (S_12a) and training of nursing staff regarding hygiene (S_12b)	Infection prevention and assessment of medical care	Medical	recommendation S2-guideline
	Access to endoscopic dysphagia diagnostic equipment (S_14)	Appropriate assessment of dysphagia and coordinated therapeutic measures	Therapeutic-rehabilitative	recommendation expert
	Offered social care/ social care used by patients (S_15)	Assessment if social participation is promoted by the nursing care facility and if it is used by the patients	Nursing	recommendation expert
	Assessment of QoL (quality of life, social participation, activity of living) (S_16)	Considering the effect of mechanical ventilation on patients' quality of life, participation in social life and daily activities)	Nursing/ Therapeutic-rehabilitative	recommendation S2k-guideline
Process indicators (n = 8)	Comprehensive transition and discharge management into long-term care (P_01)	Reduction of complications during the transition (patient level)	Medical	recommendation S2k-guideline
	Induction of informal caregivers by nursing team (P_02)	Improvement of processes in the home care setting, optimal patient care by training of relatives, increase in patients' participation in social life, support regarding prevention/reduction of concomitant diseases and complications	Nursing/ Therapeutic-rehabilitative	recommendation S2k-guideline
	Patients' say in choosing they nursing specialist and therapists (P_05)	The patient should choose their nursing specialist and therapists, to guarantee a continuous and appropriate care for the patients	Nursing	recommendation S2k-guideline
	Guaranteed supply of therapeutic measures and continuous therapy sequence (P_07)	The patient receives physiotherapy, occupational and speech therapy within a defined time period (with defined therapy sequence and without pauses)	Therapeutic-rehabilitative	recommendation expert
	Mobilisation of the patient (P_11)	Provision of activating care	Medical	recommendation S2k-guideline
	Follow-up visit of patients at the initiating weaning centre (P_14)	Optimisation of mechanical ventilation settings	Nursing	recommendation S2k-guideline
	Needs assessment by an external assessor (P_15)	Guaranteed optimal care situation	Nursing/ Therapeutic-rehabilitative	recommendation S2k-guideline
	Inter-professional cooperation (P_16)	The therapeutic concept is coordinated between all therapeutic groups; all responsible health care professions meet regularly	Therapeutic-rehabilitative	recommendation expert
Outcome indicators (n = 3)	Number of hospital admissions (O_05)	Detection of complications in the intensive care; assessment of the proportion of patients that had to be re-admissioned to a hospital due to worsening of the underlying disease or due to an incident disease	Nursing	recommendation S2k-guideline
	Complication rate (O_06)	Reporting defined complications	Medical/ Nursing/ Therapeutic-rehabilitative	recommendation S2k-guideline
	Cases for which the extend of nursing care could be reduced (O_08)	Recording of reduction of scope of nursing care	Nursing	recommendation S2k-guideline

### Development of questionnaires and pilot study

Based on the final QI set, 2 questionnaires were developed to assess the quality of care in the routine HMV care in a cross-sectional manner. The first questionnaire collects structural data about the health care facility and the second one collects process and outcome data related to the patient. A pilot study to assess the feasibility of documenting the final set of QIs in routine HMV care was carried out in five facilities between December 2019 and May 2021. All participating facilities were shared living communities (SLC) specialised in intensive care. A manual with a detailed description of the questionnaire items was provided.

The mean time to document the information was about 15 min for each questionnaire. The feedback given on both questionnaires was as follows: The facilities reported that it was feasible to complete the questionnaires in their daily routine. Their remarks were categorised in three groups: 1). term missing accuracy; 2). problem of understanding; and, 3). information not documented in patients’ files or documented elsewhere. For the structural QIs in 12 questions terms missed accuracy, for 4 questions comprehension problems occurred and 3 questions could not be answered as the information was not documented or documented elsewhere. For patient related process and outcome QIs in 2 questions terms missed accuracy and for 4 questions comprehension problems occurred. The remarks were discussed and the wording of the questionnaires were adjusted. The final questionnaires are available in the Supplement (Supplementary Material S2 and S3).

The results of the pilot study are summarised in Tables 2 and 3. An average of six patients lived in the participating institutions, of which at least two were dependent on mechanical ventilation. At least 80% of the nurses working in the facilities had documented experience working with intensive care and at least one of the nurse staff was a specialist for anaesthesia and intensive care, but in only one of the facilities all staff had an additional qualification in the field of HMV.

**Table 2:** Results of the pilot study– health care facilities

Health care facility		1	2	3	4	5
Type of health care facility^$^		SLC	SLC	SLC	SLC	SLC
Number of	Patients	8	4	7	5	6
	Invasive ventilated	2	2	2	1	0
	Non-invasive ventilated	0	0	1	4	1
	Nursing assistants	0	0	0	2	3
	Nursing specialists	20	10	17	20	17
	Respiratory therapist	1		1	0	0
	Nursing specialists for anaesthesia and intensive care	1	2	1	2	3
	Nursing specialists with professional experience in mechanical ventilation	18	10	14	13	5
	Nursing specialist with additional qualification	20	8	5	7	11
	Nursing specialists taking part in regular emergency training	20	10	17	22	17
	Mean number of nursing specialist per working shift in the facility	3	2	4	2	2
	Therapists^*^	5	2	4	3	
	Language and speech therapists^*^	1	0	1	1	
	Occupational therapists^*^	2	1	1	1	
	Physiotherapists^*^	2	1	2	1	
Participation in external quality circles		no	no	yes	yes	yes
How often in	Q1			1	1	1
	Q2			1	1	0
	Q3			1	1	1
	Q4			0	1	0
Multidisciplinary case conferences		yes, twice per year	no	yes	yes	yes, if required
How often in	Jan			0	1	
	Feb			1	1	
	Mar			0	1	
	Apr			0	1	
	May			1	1	
	Jun			0	1	
	Jul			0	1	
	Aug			1	1	
	Sep			0	1	
	Oct			0	1	
	Nov			1	1	
	Dec			0	1	
Participating professions		nurses, general practitioner, (respiratory) therapists		nurses, general practitioner, (respiratory) therapists language and speech therapists	nurses - other professions are involved if necessary	nurses, general practitioner, mechanical ventilation provider, language and speech therapist (external)
Standardised tools for early detection of complications	Documentation of defecation	yes	yes	yes	yes	yes
	Wound documentation	yes	yes	yes	yes	yes
	Temperature measurement	yes	yes	yes	yes	yes
	Documentation of vital signs	yes	yes	yes	yes	yes
	Documentation of oxygen saturation	yes	yes	yes	yes	yes
	Documentation of pain	yes	yes	yes	yes	yes
	Documentation of fear	yes	no	no	no	yes
	Documentation of hyperventilation	yes	no	yes	yes	yes
	Documentation of anomalies	yes	no	yes	yes	yes
	Other				Respiratory parameters	
SOP for handling complications		yes	no	yes	yes	yes
Contact info available		relative/ attendant, general practitioner, anaesthetist, mechanical ventilation provider	relative/ attendant, general practitioner, mechanical ventilation provider	relative/ attendant, general practitioner, anaesthetist, mechanical ventilation provider	relative/ attendant, general practitioner, anaesthetist/ pulmonologist, responsible weaning centre, mechanical ventilation provider	relative/ attendant, general practitioner, anaesthetist/ pulmonologist, mechanical ventilation provider
Evaluated emergency plan	Fire	yes	yes	yes	yes	yes
	Evacuation	yes	yes	yes	yes	yes
	Power failure	yes	no	yes	yes	yes
	Staff shortage	yes	no	yes	yes	yes
Emergency set available for every patients		yes	no	yes	yes	yes
Annual emergency training		yes	yes	yes	yes	yes
Hygiene plan/ SOP for infection prevention		yes	yes	yes	yes	yes
Nursing specialist as hygiene agent		yes	yes	yes	yes	yes
Annual training regarding hygiene		yes	yes	yes	yes	yes
Availability of an endoscope for swallowing diagnostics		no	no	no	yes	yes
Consultation for social participation		yes	no	yes	yes	yes
Offer of social participation		yes	yes	no	yes	yes
Number of day care attendants		0	1	0	2	2
If there is no day care attendant, does a nurse take on this part?		yes		yes	yes	yes
Regular assessment of quality of life		no	no	no	no	no
If yes, which instrument is used						
Number of invasive ventilated patients, that had to be transferred due to acute hospitalisation within the past 12 months		0	2	0	4	0
Number of invasive ventilated patients, that have been transferred due to acute hospitalisation within the past 12 months within a structured process		0	0	1	4	0

^$^HC = home care, SLC = shared living community/ ^*^ All therapists are extern and not directly employed by the health care facilities

**Table 3:** Results of the pilot study– HMV patients

Patient		1	2	3	4	5	6	7	8	9
Birth year		1974	-	1969	1957	1939	1956	1942	1971	1938
Sex		female	male	female	male	male	male	male	male	male
Type of health care institution^*^		HC	SLC	SLC	SLC	SLC	SLC	SLC	SLC	SLC
Invasive ventilated		x	x	x	x		x			
Non-invasive ventilated						x		x	x	x
Mechanical ventilation since		1993	2018	2015	2020	2020	2017	2021	2017	2019
Patient admission	Discharged from hospital	yes	yes	yes	yes	yes	yes	yes	yes	yes
	If yes, structured transition	no	yes	yes	yes	yes	yes	yes		yes
Training of relatives documented		no	yes	no	no	no	no	no	no	no
Documentation of patient involvement regarding	Nursing staff	no	no	no	no	no	no	no	no	yes
	Therapists	no	yes	no	no	no	yes	no	yes	yes
Therapeutic measures in the last 3 months	Physiotherapy	yes	yes	yes	yes	yes	yes	yes	yes	yes
	Speech therapy	no	yes	no	yes	yes	yes	yes	yes	yes
	Occupational therapy	no	yes	yes	yes	yes	yes	yes	yes	yes
Therapy pause (min. 3 months)		no	no	no	yes	no	no	no	no	no
Regular mobilisation of the patient		yes	yes	yes	yes	yes	n.d.	yes	yes	yes
Care assessment of patient: presentation at the responsible weaning centre within the 12 months after discharge from hospital		-	no	no	no	no	no	no	no	no
Reassessment	Nursing concept	no	yes	no	yes	yes	yes	yes	yes	yes
	Medical aid concept	no	yes	no	yes	yes	yes	yes	yes	yes
	Weaning potential	no	yes	no	yes	yes	yes	yes	yes	yes
Doctoral visit		no	yes	no	yes	yes	yes	yes	yes	no
Multidisciplinary case conferences		no	no	no	no	no	no	no	n.d.	no
Number of hospital admissions		0	1	0	2	0	0	0	0	0
Frequency of complications	Decubitus	0	0	0	2	0	0	0	0	0
	Pneumonia	0	2	0	3	2	0	0	0	0
	Unplanned change of cannula	0	2	0	0	0	0	0	0	0
Reduction of nursing care		no	no	no	no	no	no	no	n.d.	yes
If yes, to which extend		0	0	0	0	0	0	0	0	0
If no, increase		no	yes	no	yes	yes	yes	yes	yes	-

^*^HC = home care, SLC = shared living community

For the patient questionnaire, information about nine patients could be collected. One of them lived at home and the other eight in an SLC. Most of them were male and had an average age of 55 years. Almost none of the relatives of the patients was trained in HMV care. All patients received at least one type of therapy (either physical, speech or occupational therapy) and just one of them had a therapy pause for at least three months. Furthermore, the patients were mobilised regularly. None of the patients presented themselves at a weaning centre in the last year and two of them received no revision on their assessment. Two of them had to be hospitalised in the last year because of an acute complication.

## Discussion

The aim of this study was to develop a set of quality indicators (QIs) measuring the quality of HMV care using a standardised and evidence-based approach. The QI board established 26 QIs according to structure (15 indicators), process (8 indicators) and outcome (3 indicators). The developed QIs set cover different relevant aspects of long-term HMV care. Previous publications reported the issues and challenges targeted by our QIs set, for instance the importance of care based on the patients’ wishes, caregiver education and ongoing assessment of skills [13, 30]. For the QI development, the QI board chose the Donabedian concept as theoretical framework. Our QI set fulfils all predefined methodological requirements.

Prior QIs for the care of HMV patients were developed focusing on long-term oxygen therapy [31] or were drafted more generally, stating requirements for long-term care management (i.e. necessary infrastructure, ventilation criteria and applicable service standards) [32]. A previous study exclusively targeting the transition from in-hospital ventilation to long-term HMV care in Germany developed a set of 10 QIs using a different approach [33]. Based on a targeted process chain and a literature review their QIs were developed and consented by an expert committee including industrial, economic, and medical experts but no patient representatives.

The first structural indicator in our QI set comprises the comprehensive transition management to a weaning centre (S_01). A comprehensive transition management requires a certified weaning centre, a defined registration process, the submission of medical records from the intensive care unit, a transition protocol, a discharge letter, and regular contact to the responsible weaning centre. This checklist should reduce complications within the transition process. As we wanted to develop a set of QIs that can be assessed in routine long-term care, we focused in the QI S_01 on the completeness of the necessary documents from the transferring clinic.

As our QIs set especially targets the long-term HMV care after the discharge process, we set a huge focus on the qualifications for HMV care obtained by the nursing care facilities. In a previous long-term observational study covering different regions in Germany, needs and necessary structures for HMV care were identified [34]. It was stated that information on the range of services, on quality preserving or improving measures (e.g. qualification standards for the employees or certificates of the health care providers) and on (additional) qualification of the nursing specialists is scarce [34]. To date, nationwide and binding admission criteria for the care of HMV patients for health care providers have not been established [35]. We tried to cover these aspects in the structural indicators qualification of nursing staff (S_05) and (additional) qualification of therapists (S_07). Furthermore, we considered the participation of the nursing care facility in external quality circles as a structural indicator in our QI set (S_10). Quality circles are a recognised and proactive quality assurance method. Their goal is to identify potential problems and to develop suggestions for improvement for a more patient- and need-oriented care.

Another focus of our structural QI set is patient safety. We targeted patient safety in the QIs acute-hospitalisation of patients (S_02), emergency management and concepts in case of infrastructure deficiencies (S_03), early detection of complications (S_04a) and complication management (S_04b), hygiene plan and concept (S_12a) and training of nursing staff regarding hygiene (S_12b), and assessment of QoL (quality of life, participation, activity preservation) (S_16). Although different projects to strengthen safety awareness in long-term care exist [36–39], such systems are only partly implemented in the home care setting. We included a QI to assess if case conferences were regularly performed and documented (S_11). Hence, critical incidents in long-term care are usually discussed in (interdisciplinary) case conferences.

Regarding the process indicators, we firstly included the comprehensive transition and discharge management to the long-term care sector (P_01) at patient level. This transition phase between in-hospital and long-term care is very vulnerable. Hence, an exhaustive organisation by the discharging hospital is necessary. The QI P_01 comprises several requirements for this discharge process (e.g. stable disease, or supply with all necessary medical devices and aids) as well as a checklist based on the DNQP expert standard for discharge management in nursing care. The QI P_07 covers the guaranteed supply of therapeutic measures and continuous therapy sequence. A S2k guideline for invasive and non-invasive home mechanical ventilation, recommends physio-, occupational and speech therapy for all HMV patients [2]. These measures are individually prescribed by the treating physician depending on the indication, his/her own judgement, and on budgetary and bureaucratic decisions [40]. However, home visits by the treating physician are seldom made and it is not always possible for the HMV patient to visit the responsible physician in person. Hence, the assessment of patients by the treating physician is often hindered. Moreover, patients or their relatives have to apply for the therapeutic measures as “measures outside of the norm” to be reimbursed by their health care insurance [40, 41]. This can lead to pauses or discontinuation of the therapeutic measures. As mechanical ventilation is not part of the diagnostic catalogue justifying “special prescription requirement”, physicians are not able to prescribe a therapy without any further explanation and (sometimes) off-budget. Additionally, the lack of professional therapists is a problem concerning all health care sectors [22, 42].

Different professions are involved in HMV care (i.e. physicians, nursing specialists, therapists, medical device providers, and cost units) and complex medical services require regular interdisciplinary consultations to avoid adverse outcomes [2, 35]. For this reason, we included the QI P_16, which covers the inter-professional cooperation in planning and re-assessment of the therapeutic concept. These aspects of individualised planning of a multimodal therapeutic concept and nursing care were also targeted in a previous study [33], however a detailed description of therapeutic and nursing care measures is missing.

The annual reassessment of HMV patients regarding their weaning potential is recommended [2]. The reassessment by an external assessor is performed in cooperation with the Medical Service (MD). We defined two QIs targeting the re-presentation of patients at the responsible weaning centre (P_14) and the reassessment of patients by an external assessor (P_15) regarding the nursing care concept, the medical device and aids concept, and the weaning potential, respectively. Therefore, both reassessment options are covered in our QI set.

Patients’ involvement in health care decision making is another important aspect. Appropriate care should be based not only on medical requirements but also on the patient’s wishes and needs [2], and we covered this aspect by the process indicator P_05. Additionally, we considered the necessity that the patient has to be informed on nursing care and treatment options and also the patient’s will as a necessary structure. That has to be considered in the structural QI acute-hospitalisation of patients (S_02). As this information is not routinely documented in the patient records of nursing facilities, it was not possible to include these aspects as process indicators.

Concerns about patient autonomy, particularly in light of recent discussions about the IPReG, highlight the relevance and necessity of QIs focusing on social participation and shared medical decision making of patients. This was the subject of one process QI regarding patients’ say in choosing their nursing specialist and/ or therapists (P_05) and two structural QIs regarding the offer of social care and social participation by the responsible nursing facility (S_15) and the assessment of quality of life (S_16). In the first draft of the QI set after the first workshop, even more QIs covering these aspects were included e.g. on patients’ power of attorney and living will or on activity preservation and social participation. However, one important requirement for the review and final consent was that the necessary information for patient-related (process and outcome) QIs should be documented in the patient file and thereby easily assessable. For the above-mentioned points, to date no routine documentation in nursing care facilities exists. Hence, the respective QIs were not included in the final set.

Eventually, our first outcome QI comprises the number of hospital admissions (O_05). The goal is to evaluate potential complications in long-term HMV care, as the care situation of these patients is often unstable. Emergency interventions and repeated hospitalisations due to worsening of the underlying disease or due to an incident disease are frequently necessary. Our second outcome indicator targets the complication rate (O_06). In HMV patients potentially life-threatening situations can arise at any time. Hence, preventive measures are of high importance. These measures include the documentation of complications such as constipation, decubitus, and pneumonia. Our third outcome indicator covers the reduction of nursing care (O_08). If the general condition of an HMV patient improves, the care delivered by nursing specialists can be reduced. By training the patient and his/her relatives, they are able to take over some of the nursing care tasks. This is an important aspect in long-term HMV care, as it enhances the patients’ autonomy.

Outcome indicators are underrepresented in our QI set, because they are more difficult to assess and it is difficult to adjust for potential factors influencing the outcome due to heterogeneity of HMV patients. Most of our QIs measure the structure of care. These structural components reflect the system and setting in which care is delivered i.e. educational and training activities of the nursing staff, appropriateness of the equipment and organisational measures. The advantage of structural QIs is their expediency as they can be assessed easily and inexpensively [43, 44]. They are strongly related to outcome indicators, which are more seldom documented in the routine care of HMV patients. By embedding the structural QIs in the questionnaire for the health care facility we indirectly addressed the necessary processes to guarantee the performance quality.

To test the feasibility of the developed questionnaires we conducted a prospective pilot study. Their implementation in the care setting was considered as practicable and retrieved plausible results for all institutions and patients. Furthermore, all facilities reported that it was possible to complete the questionnaires in their daily routine in a rather short amount of time. The QI set is an instrument that could influence the quality of care of HMV patients by surveying health care delivered. They can facilitate transferring evidence into routine nursing care. In our pilot study, we could collect information about 9 patients and 5 SLC, which was sufficient to test the feasibility of the questionnaires, but not designed to give first hints in long-term HMV care.

### Strengths and limitations

Our QI set development process has strengths and limitations. We decided to develop a set of QIs using a standardised, evidence-based approach following previously defined recommendations to ensure high methodological quality and maximal transparency of our results [24, 25]. The set of indicators was developed by a multidisciplinary board to guarantee a wide acceptance of the results by institutions and organizations engaged in HMV care in Germany. Unfortunately, there is no register of HMV patients in Germany. HMV patients living at home are difficult to locate, as they might organise long-term care services by themselves without using a health care provider. The involvement of the HVM patient representative, who is also active in different HMV and long-term care patient organisations, was therefore particularly important to the development of our QI set. She has first-hand knowledge on the individual and very heterogeneous health care situations of HMV patients as they differ not only regarding their medical diagnoses but also in the areas and intensity of need for support. The structural QIs (Supplementary Material S3) cannot cover the situation of patients living at home as it was developed to evaluate the care situation in facilities providing long-term care, but the quality of long-term care service provided to HMV patients by shared living communities and specialised long-term care nursing services can be assessed. Additionally, information on HC is covered in the patient-related QIs (Supplementary Material S2).Our questionnaires allow a follow-up of both patients and nursing facilities as we included an entry field for a patient and a facility ID, respectively. However, an official database, for example integrated within the MD Bavaria, would be necessary to collect and store the follow-up data. A limitation in our QI development is the varying evidence base due to a lack of published high level evidence studies. Therefore, a significant proportion of the developed indicators is based on a S2k-guideline or on expert opinions. Furthermore, it has been discussed that the Donabedian concept is not sufficiently differentiable for questions in nursing care [45]. However, by extending the concept by the three health care domains medical care, nursing care and therapeutic-rehabilitative care, a more detailed itemization for the quality of care was possible.

## Conclusion

The QIs reflect the actual status of evidence and appear to reflect the reality of the care situation among HMV patients. Furthermore, they should meet the demands made within the IPReG. The development of standardised evidence-based QIs to evaluate HMV care is a step towards implementing a standardised quality assurance program for HMV, which could improve the quality of care of these patients. Due to the heterogeneity of the HMV patient population, bigger studies are needed to confirm the relevance of the individual aspects. This also applies for the implementation of the set of QIs and the QI questionnaires.

### Electronic supplementary material

Below is the link to the electronic supplementary material.

Supplementary Material 1

Supplementary Material 2

Supplementary Material 3

## Data Availability

The datasets used and/or analysed during the current study are available from the corresponding author on reasonable request.
