# Supplementary material for: Development and pilot-testing of an evidence-based quality indicator set for home mechanical ventilation care: the OVER-BEAS project
Source: BMC Health Serv Res. 2024 Jan 30;24:152. doi: 10.1186/s12913-024-10583-2 (PMC10829274; doi:10.1186/s12913-024-10583-2)
Supplement: Supplementary file 1 — Supplementary Material 1 [file 12913_2024_10583_MOESM1_ESM.docx]

**Table S1: Comparison of the initial QI set with the final QI set.**

| **Phase** | **Initial set of quality indicators (n=40)** | **Final set of quality indicators (n=26)** |
| --- | --- | --- |
| **Structural indicators** | | |
| *Medical care* | Comprehensive transition management to weaning centre (MV_QI_01a) | Comprehensive transition management to weaning centre (from hospital)/ contact to weaning centre/ contact person at weaning centre (S_01) |
|  | Case conferences (MV_QI_02) | Interdisciplinary case conferences (S_11) |
|  | Transition management/ emergency management/ hospitalisation (MV_QI_03) | Acute-hospitalisation of patients (S_02) |
|  | Availability in case of emergencies (MV_QI_05) | Emergency management and concepts in case of infrastructure deficiencies (S_03) |
|  | Hygiene plan and concept (MV_QI_07) | Hygiene plan and concept (S_12a) and training of nursing staff regarding hygiene (S_12b) |
|  | Surveillance/ medication plan (MV_QI_08) |  |
|  | Ethics/ palliation: patient decree, DNR recommendation (reanimation status), power of attorney (MV_QI_09) |  |
|  | *[process indicator PV_QI_07 and outcome indicators TRV_QI_14 and TRV_QI_15]* | Offered social care/ social care used by patients (S_15) |
|  |  | Early detection of complications (S_04a) and Complication management (S_04b) |
| *Therapeutic-rehabilitative care* | Additional qualifications of therapists (TRV_QI_01) | Qualification of nursing staff (basic and additional qualifications) (S_05) |
|  | Establishment of a security concept and regular maintenance of mechanical ventilation devices (TRV_QI_02) |  |
|  | Employment of registered nursing staff (TRV_QI_03) | Qualification of nursing staff (basic and additional qualifications) (S_05) |
|  | Access to instrumental swallowing diagnostic (TRV_QI_04) | Access to instrumental swallowing diagnostic (S_14) |
|  |  | Qualification and additional qualification of therapists (S_07) |
| *Therapeutic-rehabilitative and nursing care* | *[Outcome indicator TRV_QI_13]* | Assessment of QoL (quality of life, participation, activity preservation) (S_16) |
| *Nursing care* | Qualification of nursing staff (basic and additional qualifications) (PV_QI_01) | Qualification of nursing staff (basic and additional qualifications) (S_05) |
|  | Nursing staff to patient ratio (PF_QI_02) | Nursing staff to patient ratio (ambulatory intensive care - assisted living communities) (S_06) |
|  | Participation of the nursing facility in external quality circles (PV_QI_03) | Participation of the nursing facility in external quality circles (S_10) |
|  | Personnel continuity in the nursing facilities (PV_QI_04) |  |

**Table S1 (continued).**

| **Process indicators** | | |
| --- | --- | --- |
| *Medical care* | Blood gas analysis (MV_QI_06) |  |
|  | Determination of mobilisation of the patient (MV_QI_11) | Determination of mobilisation of the patient (P_11) |
|  | Comprehensive transition and discharge management to the ambulatory sector (MV_QI_01b) | Comprehensive transition and discharge management to the ambulatory sector (P_01) |
| *Therapeutic-rehabilitative care* | Guaranteed supply of therapeutic measures (TRV_QI_05) | Guaranteed supply of therapeutic measures and continuous therapy sequence (P_07) |
|  | Adjustment of the environment (TRV_QI_06) |  |
|  | Continuous therapy sequence (TRV_QI_07) | Guaranteed supply of therapeutic measures and continuous therapy sequence (P_07) |
|  | Inter-professional cooperation (TRV_QI_08) | Inter-professional cooperation (P_16) |
|  | Instruction of relatives by the nursing team (TRV_QI_09) | Instruction of relatives by the nursing team (P_02) |
|  | Activity with devices and medical aids (TRV_QI_10) |  |
| *Nursing care* | Offered social care/ social care used by patients (PV_QI_07) | *[structure indicator S_15]* |
|  | Re-presentation of patients at the responsible weaning centre (PV_QI_09) | Re-presentation of patients at the responsible weaning centre (P_14) |
|  | Patients' say in choosing they nursing specialist (PV_QI_10) | Patients' say in choosing they nursing specialist and therapists (P_05) |
|  | Determine if the patient was assessed by an external assessor (PV_QI_11) | Determine if the patient was assessed by an external assessor (P_15) |
|  | Information of the patient regarding different options in care and therapy and consideration of patient's wishes (PV_QI_12) |  |
|  | Assessment if the patient was mobilised (PV_QI_13) | *[included in process indicator P_11]* |
| **Outcome indicators** | | |
| *Medical care* | Complications during emergencies (MV_QI_04) | Complication rate (O_06) |
| *Therapeutic-rehabilitative care* | Weaning potential (TRV_QI_11) | *[included in process indicator P_14]* |
|  | Prevention of pneumonia (TRV_QI_12) | *[included in structure indicator S_12a and S_12b]* |
|  | Quality of life (TRV_QI_13) | *[structure indicator S_16]* |
|  | Participation in social life (TRV_QI_14) | *[included in structure indicator S_15]* |
|  | Activity preservation (TRV_QI_15) | *[included in structure indicator S_15]* |
| *Nursing care* | Number of hospital admissions (PV_QI_17) | Number of hospital admissions (O_05) |
|  | Occurrence of decubitus (PV_QI_19) | Complication rate (O_06) |
|  | Necessity of cannula exchange (PV_QI_20) | Complication rate (O_06) |
|  | Cases for which the extend of nursing care could be reduced (PV_QI_21) | Cases for which the extend of nursing care could be reduced (O_08) |
