# Supplementary material for: Development and pilot-testing of an evidence-based quality indicator set for home mechanical ventilation care: the OVER-BEAS project
Source: BMC Health Serv Res. 2024 Jan 30;24:152. doi: 10.1186/s12913-024-10583-2 (PMC10829274; doi:10.1186/s12913-024-10583-2)
Supplement: Supplementary file 2 — Supplementary Material 2 [file 12913_2024_10583_MOESM2_ESM.docx]

**Supplementary Material S2: Survey form OVER-BEAS – patient-related quality indicators**

*This questionnaire was developed and tested in German and the following translation has not been validated.*

Date of the survey (DD/MM/YYYY)

Patient ID

Year of birth (YYYY)

Gender:

*Male/ Female/ Diverse*

Type of nursing care facility:

*Shared living community/ Nursing home/ Outpatient care service (home care)*

Supply situation:

*Invasive ventilation since (YYYY)/ Non-invasive ventilation since (YYYY)*

1. **Patient Admission**
   1. Was the patient transferred from a hospital to your facility?

*Yes/ No/ Not documented*

- - 1. If yes, was the transition structured (1) ?

*Yes/ No/ Not documented*

1. **Training of Relatives**
   1. Is a training (2) of relatives/informal caregivers documented?

*Yes/ No*

1. **Documentation regarding the right of patients to have their say**
   1. Is the say regarding the choice of nursing specialists documented?

*Yes/ No*

- 1. Is the right to have a say in the choice of therapists documented?

*Yes/ No*

1. **Care for patients**
   1. Has the patient received one or more of the following therapeutic measures **in the last 3 months**?
      1. Physiotherapy

*Yes/ No/ Not documented*

- - 1. Speech therapy

*Yes/ No/ Not documented*

- - 1. Occupational therapy

*Yes/ No/ Not documented*

- 1. Was the therapy discontinued (at least 3 months)?

*Yes/ No/ Not documented*

- 1. Was the patient mobilised regularly (at least 1-2 times per day for 2 hours) (3)?

*Yes/ No/ Not documented*

1. **Assessment/evaluation of patients**
   1. Did the patient present him/herself at least once at the responsible respiratory centre within 12 months after discharge from hospital?

*Yes/ No/ Not documented*

- 1. Has the patient been reassessed **within 12 months** of discharge from hospital for one or more of the following aspects?
     1. The nursing care concept

*Yes/ No/ Not documented*

- - 1. The Assistive Technology Concept

*Yes/ No/ Not documented*

- - 1. The Weaning Potential

*Yes/ No/ Not documented*

- 1. Has a doctor's visit with special expertise in intensive care and respiratory medicine taken place at least **once per quarter** in the last calendar year?

*Yes/ No/ Not documented*

- 1. Did a multidisciplinary case conference take place **once per quarter**, in which the therapeutic concept for the patient was agreed?

*Yes/ No/ Not documented*

1. **Inpatient admissions**

Number of inpatient admissions (4) of the patient **within the last 12 months**.

1. **Complications**
   1. How often did the following complications occur per quarter in the last calendar year?
      1. Pressure ulcer

*Q1/ Q2/ Q3/ Q4*

- - 1. Pneumonia

*Q1/ Q2/ Q3/ Q4*

- - 1. Unplanned cannula change

*Q1/ Q2/ Q3/ Q4*

1. **Reduction of the scope of care(**
   1. Was the scope of nursing care reduced due to an improvement of the general condition in the last calendar year?

*Yes/ No/ Not documented*

- - 1. If yes, to what extent

1. *hour/ 2 hours/ 3 hours/ 4 hours/ ≥5 hours*
   - 1. If not, was the scope of nursing care increased (due to a deterioration of the general condition)?

*Yes/ No/ Not documented*

*Filling in instructions*

1. *Definition of a structured transition based on the DNQP expert standard "Discharge management in nursing":*

- *Stability of underlying and concomitant disease(s)*
- *Type of ventilation access, ventilation mode, duration of ventilation, or duration of possible spontaneous breathing phases must be known*
- *Oxygen flow rate must be known*
- *Secretion management measures are in place*
- *Technical equipment for ventilation and monitoring incl. accessories are available*
- *Assumption of costs for the provision of care and aids has been granted*
- *Provision of the patient with all necessary equipment, aids and materials, and presence of these aids at the time of discharge*
- *Appointment for the first follow-up examination in a weaning centre has been made*
- *Discharge medication incl. medication on demand is determined*
- *Screening for multi-resistant pathogens was carried out*
- *The form of housing and nursing care is determined, including an examination of the home with regard to accessibility and counselling with regard to constructional or organisational/structural adaptation of the home conditions if necessary, on the basis of "housing improvement measures"*
- *The scope of care (attendance times of care), time frame and contents of the care measures is defined*
- *Therapeutical measures i.e. physiotherapy, speech therapy and occupational therapy are planned*
- *Training of patient and relatives has taken place*
- *Psychosocial support for the patient and, if necessary, for the relatives is planned*

1. *Training of relatives, for example, on transfer, as well as everyday tasks (e.g. positioning, personal hygiene, eating) and home activities (e.g. mobility, leisure activities).*
2. *Definition of mobilisation according to the DNQP expert standard "Maintenance and promotion of mobility in in nursing care":*

*All activating measures that promote the patient's mobility or ability to move, i.e. the physical ability to move independently over short distances (with aids if necessary), the patient's physical ability to move independently over short distances (if necessary with aids) and to change the position of the body independently. Through mobilization, it is also intended to prevent secondary diseases such as pneumonia (pneumonia), pressure ulcers or joint contractures.*

1. *Inpatient admissions due to a worsening of the underlying disease or an additional disease*
