# Supplementary material for: Development and pilot-testing of an evidence-based quality indicator set for home mechanical ventilation care: the OVER-BEAS project
Source: BMC Health Serv Res. 2024 Jan 30;24:152. doi: 10.1186/s12913-024-10583-2 (PMC10829274; doi:10.1186/s12913-024-10583-2)
Supplement: Supplementary file 3 — Supplementary Material 3 [file 12913_2024_10583_MOESM3_ESM.docx]

**Supplementary Material S3: Survey form OVER-BEAS – structural quality indicators**

*This questionnaire was developed and tested in German and the following translation has not been validated.*

Date of the survey (DD/MM/YYYY)

Centre ID

Type of nursing care facility:

*Shared living community/ Nursing home/* Specialised long-term care nursing service *(home care)*

Please complete this survey form for the calendar year (YYYY)

1. **Residents**
   1. Number of residents in your facility/nursing service
   2. Number of invasive ventilated patients in your facility/nursing service
   3. Number of non-invasive ventilated patients in your facility/nursing service
2. **Nursing staff (1)**
   1. Number of employed nursing assistants (2) in your facility/nursing service
   2. Number of employed nursing specialists (3) in your facility/nursing service

Additionally for nursing specialists with a foreign qualification:

- 1. Number of registered nursing specialists with a degree obtained abroad
  2. Number of registered nursing specialists with a degree recognised in Germany
  3. Number of employed nursing staff in your facility who have one or more of the following additional qualifications:
     1. Respiratory therapists
     2. Nursing specialists for anaesthesia and intensive care
     3. Nursing specialists with professional experience in the field of ventilation (at least 1 year in the last 5 years)
     4. Nursing specialists with additional qualification (4)
     5. Nursing specialists who have participated in regular emergency training, including training on emergency tracheostomy tube exchange, in the last year
  4. Average number of nursing specialists on site per work shift
  5. Number of nursing specialists in training in your institution (optional)

1. **Therapists (5)**
   1. Number of therapists employed in your care facility:
      1. Speech therapists
      2. Occupational therapists
      3. Physiotherapists
   2. Do you check whether the therapists employed in your care facility have a license to use the professional title or a qualification recognised in Germany?

*Yes/ No*

- 1. Number of therapists in your care facility with permission to use the professional title and a qualification recognised in Germany:
     1. Speech therapists
     2. Occupational therapists
     3. Physiotherapists
  2. Number of therapists in your care facility who have an additional qualification or experience in the field of intensive care:
     1. Speech therapists
     2. Occupational therapists
     3. Physiotherapists
  3. Number of external therapists working in your care facility:
     1. Speech therapists
     2. Occupational therapists
     3. Physiotherapists

1. **External quality circles**
   1. Does the management and/or do the employees of your institution participate in external quality circles for further training?

*Yes/ No*

- - 1. **If yes**, how often: (enter number per quarter)

*Q1/ Q2/ Q3/ Q4*

1. **Multidisciplinary case conferences**
   1. Are multidisciplinary case conferences held on ventilated patients?

*Yes/ No*

- - 1. **If yes**, how often: (enter number per month)

*Jan/ Feb/ Mar/ Apr/ May/ Jun/ Jul/ Aug/ Sep/ Oct/ Nov/ Dec*

- 1. Which professions are involved?
     1. Nursing Specialists
     2. General practitioners
     3. Respiratory therapists
     4. Pneumologists
     5. Device providers
     6. Payors *(Kostenträger)* of out-of-hospital care
     7. Speech therapists

*internal/ external*

- - 1. Occupational therapists

*internal/ external*

- - 1. Physiotherapists

*internal/ external*

- - 1. Other

1. **Early detection and management of complications**
   1. Are standardised tools used for early detection of complications?
      1. Documentation of bowel movements

*Yes/ No*

- - 1. Wound documentation

*Yes/ No*

- - 1. Temperature measurement

*Yes/ No*

- - 1. Documentation of vital signs *(blood pressure, pulse, body temperature, neurological [with pupillary reaction if applicable], respiratory rate if applicable)*

*Yes/ No*

- - 1. Oxygen saturation documentation

*Yes/ No*

- - 1. Documentation of pain

*Yes/ No*

- - 1. Documentation of fear

*Yes/ No*

- - 1. Documentation of hyperventilation

*Yes/ No*

- - 1. Documentation of anomalies

*Yes/ No*

- - 1. Other (*e.g. cerebral seizure protocol; fixation protocol; sleep protocol)*

*Yes/ No*

- 1. Does your facility have standardised work instructions for dealing with complications? *(e.g. dislocation/ misplacement of the tracheal cannula, saturation decline, etc.)*

1. **Emergency management and concepts in case of impaired infrastructure (7)**
   1. From which individuals are the contact details available? Are they verified on a regular basis (8)?
      1. Relative/ (legal) carer
      2. Responsible general practitioner/family doctor
      3. Responsible pneumologist/anaesthetist
      4. Responsible Weaning Centre
      5. Ventilator device provider
   2. For which situation is an evaluated emergency plan available?
      1. Fire
      2. Evacuation *(e.g. in case of floods)*
      3. Power failure
      4. Staff shortfall/ substitution plan
   3. Is an emergency kit (9) available for **each** patient at all times?

*Yes/ No*

- 1. Does your institution carry out emergency training with the staff at least **once a year**?

*Yes/ No*

1. **Hygiene concept**
   1. Is there a hygiene plan/ Standard Operating Procedure for infection prevention?

*Yes/ No*

- 1. Is a nursing specialist appointed as hygiene representative?

*Yes/ No*

- 1. Are annual training courses on hygiene held?

*Yes/ No*

- - 1. **If yes**, does external staff participate?

*Yes/ No*

1. **Access to dysphagia diagnostic equipment**

Is an endoscope available for swallowing diagnostics?

*Yes/ No*

1. **Social care**
   1. Are patients advised on social participation

*Yes/ No*

- 1. Is social care offered to patients?

*Yes/ No*

- 1. Are everyday caregivers employed for the patients?
     1. **If yes**, how many?
     2. **If no**, does a nursing specialist perform this task?

*Yes/ No*

1. **Quality of life**
   1. Is there a regular (10) assessment of quality of life, participation and/or activity maintenance for invasive or non-invasive ventilated patients?

*Yes/ No*

- - 1. **If** **yes**, which assessment tools are used?

1. **Hospitalisation of patients**
   1. Number of invasive ventilated patients who had to be transferred due to acute hospitalisation (11) **within the last 12 months**
   2. Number of invasive ventilated patients with a structured transition (12) into hospital for acute hospitalisation **within the last 12 months**

*Filling in instructions*

1. *All questions refer to nurses in your care facility who care for ventilated patients in the home environment.*
2. *A nursing assistant has training as a "nurse's assistant" (120 teaching units of theory (one teaching unit corresponds to 45 minutes) and 80 hours of practical training).*
3. *A nursing specialist has a 3-year training in nursing care, child health and as paediatric nurse or geriatric nurse.*
4. *Additional qualification in the form of full participation in a certified course to become a "nursing specialist for out-of-hospital ventilation" (structured, in-service training of at least 120 hours). Such courses must be quality assured through certification by professional associations and must at least meet the content requirements of the DIGAB e.V. (cf.* [*https://digab.de/digab-kurse/*](https://digab.de/digab-kurse/)*).*
5. *All questions refer to therapists in your care facility who treat ventilated patients in the home environment.*
6. *The guideline "Non-invasive and invasive ventilation as a therapy for chronic respiratory insufficiency" recommends that physicians (general practitioners and/or other specialists), an out-of-hospital nursing team (specialist nurses / assistants), equipment providers for the provision of prescribed aids and their technical control, an out-of-hospital therapeutic team (speech therapy, occupational therapy, physiotherapy, social pedagogues, educationalists, psychologists) and funding agencies should be involved in the out-of-hospital care of an out-of-hospital ventilated patient. Those involved in the care of out-of-hospital ventilated patients should regularly exchange information about the health and care situation of the patients in case conferences.*
7. *Impaired infrastructure is defined as an emergency situation, e.g. fire, need for evacuation (e.g. flood), power failure or unexpected shortfall of staff.*
8. *At least every six months*
9. *Contents of an emergency kit: Ambu bag with matching mask, a 1 no. smaller tracheal cannula, a blocking syringe, a tracheal cannula spreader and a stethoscope.*
10. *At least quarterly*
11. *Acute hospitalisation is defined as a transfer from an outpatient facility to hospital due to an acute illness or deterioration in health.*
12. *A transition is considered structured if:*

- *The patient file and the ventilation folder are immediately available/viewable;*
- *A transfer sheet is prepared;*
- *The living will is available/viewable;*
- *An explanation of care and treatment options and consideration of the patient's will has taken place;*
- *An adapter for the DIN holder for the ventilator (for transport in the ambulance) is available, and;*
- *An accompanying person who knows the patient and has been instructed in the use of the ventilator is available.*
